# Carbapenem‐Resistant Enterobacteriaceae Bloodstream Infections in Neonates: Clinical Characteristics and Antimicrobial Therapy

**DOI:** 10.1155/cjid/5544605

**Published:** 2025-12-20

**Authors:** Zelei Liu, Xingpu Xu, Sanni Li, Mei Li, Jiancheng Jiao, Yinghui Guo, Li Ma

**Affiliations:** ^1^ Department of Neonatology, Children’s Hospital of Hebei Province, Shijiazhuang, China, hbpch.com; ^2^ Hebei Clinical Research Center for Pediatric Health and Diseases, Shijiazhuang, China; ^3^ Department of Pharmacy, Children’s Hospital of Hebei Province, Shijiazhuang, China, hbpch.com; ^4^ Clinical Laboratory, Children’s Hospital of Hebei Province, Shijiazhuang, China, hbpch.com

**Keywords:** antimicrobial resistance, carbapenem-resistant Enterobacteriaceae, ceftazidime/avibactam, mortality, neonatal sepsis

## Abstract

**Aim:**

To analyze the clinical characteristics, antimicrobial therapies, and outcomes of neonates with carbapenem‐resistant Enterobacteriaceae (CRE) bloodstream infections.

**Methods:**

This single‐center retrospective study included 22 neonates with CRE bloodstream infections at a tertiary children’s hospital in China (September 1, 2019–August 31, 2024). Data of clinical characteristics, risk factors, pathogens, carbapenemase testing, antimicrobial therapy, and outcomes were analyzed.

**Results:**

All 22 neonates had hospital‐acquired late‐onset infections (males, 77.3%; preterm infants, 81.8%). Prior to onset, 72.7% of the neonates had a history of broad‐spectrum antibiotic exposure. *Klebsiella pneumoniae* was the predominant pathogen (91.9%). Eighteen isolates underwent carbapenemase testing, with KPC being the most prevalent carbapenemase (66.7%). The antimicrobial agents were changed for 8 infants based on the carbapenemase testing results. Six neonates with KPC‐producing CRE infections received ceftazidime‐avibactam (CAZ‐AVI), and two neonates with NDM‐producing CRE infections were treated with CAZ‐AVI plus aztreonam. All 20 neonates who completed full treatment achieved clinical cure, while two neonates succumbed to infection before effective therapy initiation. The all‐cause mortality rate was 13.6%.

**Conclusion:**

Neonates with immature immunity are more susceptible to CRE bloodstream infections, but clinical cure could be achieved after effective antimicrobial therapy. Carbapenemase testing plays a crucial role in the decision making on treatment of neonatal CRE infections.

**Summary**

• Carbapenem‐resistant Enterobacteriaceae (CRE) pose a major threat to immunologically immature neonates. We analyzed 22 neonates with CRE bloodstream infections (BSIs) and found that 81.8% were preterm infants; 72.7% had a history of broad‐spectrum antibiotic exposure. Most (91.9%) isolates were *Klebsiella pneumoniae. K. pneumoniae* carbapenemase was the most prevalent carbapenemase (66.7%). All of the neonates who received full treatment achieved a clinical cure. Carbapenemase testing plays a crucial role in the decision making on treatment of neonatal CRE BSIs. Precise anti‐infective therapy can significantly improve the prognosis of these neonates.

## 1. Introduction

Antimicrobial resistance has significantly increased morbidity and mortality, with 4.71 million deaths globally attributed to bacterial antimicrobial resistance in 2021 [1]. Carbapenem‐resistant Enterobacteriaceae (CRE), critical priority pathogens [2], pose a major public health threat. Neonates are highly vulnerable to CRE infections due to their immunological immaturity [3]. According to data from South Asia, carbapenem resistance rates of *Escherichia coli* and *Klebsiella pneumoniae* causing neonatal sepsis were 8.1% and 10.4%, respectively [4]. Data from Chinese surveillance network have consistently reported higher CRE detection rates in neonates compared to older children [3].

Current antimicrobial regimens for CRE infections encompass both traditional antibiotics and novel agents [5–7]. However, therapeutic decision making for neonates is challenging due to the risks of traditional antibiotics [8] and the limited clinical experience with novel agents. These limitations reduce treatment options and increase mortality. Among Chinese preterm infants with carbapenem‐resistant *K. pneumoniae* (CRKP) bloodstream infections (BSIs), the reported all‐cause mortality rate was 27.1% (2015–2022) [9].

This study analyzed the clinical characteristics and outcomes of neonates with CRE BSIs with a focus on antimicrobial therapy and the epidemiology of carbapenemase. It aimed to provide a basis for optimizing antimicrobial stewardship in neonatal CRE BSIs and reducing adverse outcomes.

## 2. Patients and Methods

### 2.1. Study Design

This is a single‐center, retrospective, observational study conducted in the neonatal intensive care unit (NICU) of the Children’s Hospital of Hebei Province, Shijiazhuang, China. The NICU is a tertiary referral center that admits more than 2000 neonates annually.

Neonates with blood culture–proven CRE BSIs were enrolled. Blood culture contaminants, as determined by clinicians and microbiologists, were excluded.

The following clinical data were retrospectively collected: (1) perinatal and demographic characteristics: onset age, sex, gestational age, birth weight, mode of delivery, multiple births, and maternal comorbidities (hypertension in pregnancy and gestational diabetes mellitus); (2) risk factors: pre‐onset antibiotic exposure and invasive procedures (endotracheal intubation, central venous catheterization, parenteral nutrition, and surgery); (3) microbiological results: culture results from blood and other sites, antimicrobial susceptibility testing, and carbapenemase testing; (4) antimicrobial therapy (types, dosage, and duration); and (5) predischarge outcomes: complications (septic shock, necrotizing enterocolitis, intraventricular hemorrhage, intracerebral hemorrhage, and hydrocephalus) and death before discharge. Neonates withdrawn from treatment were followed up for 1 week after discharge to assess outcomes.

In the Chinese expert consensus for the treatment of CRE, antimicrobial agents such as polymyxins, tigecycline, fosfomycin, semisynthetic tetracyclines, and aminoglycosides are recommended [7]. However, research on these agents in neonates is limited, with all being off‐label agents. In this study, the antimicrobial therapy regimens were guided by a clinical pharmacist and present expert consensus. Third‐generation cephalosporins were used as empirical therapy for late‐onset Gram‐negative sepsis according to the Chinese Expert Consensus on Neonatal Sepsis Diagnosis (2019), while carbapenems were initiated when multidrug‐resistant (MDR) pathogens were suspected [10]. After identifying the pathogen as CRE, antimicrobial regimens involving high‐dose extended‐infusion meropenem or combination with polymyxin B were administered pre‐2020. Since 2020, carbapenemase testing has been conducted for CRE. Post‐January 2020, clinicians have made therapeutic decisions by referring to the results of carbapenemase testing. Single‐agent or combination therapy with the following agents—aztreonam, polymyxin B, or ceftazidime‐avibactam (CAZ‐AVI)—was administered in neonates who demonstrated clinical nonresponse to empirical therapy or when the meropenem minimum inhibitory concentration (MIC) of isolate exceeded 8 mg/L [7].

### 2.2. Definitions

CRE were defined as Enterobacteriaceae resistant to any one of the carbapenems. Antimicrobial susceptibility testing results were interpreted according to the latest Clinical and Laboratory Standards Institute M100 Performance Standards for Antimicrobial Susceptibility Testing [11]. Definitive antimicrobial therapy was defined as the initiation of antibiotics following pathogen identification and susceptibility testing [12]. Clinical cure was defined as the disappearance of infection signs and symptoms (such as temperature instability, hypotension, poor perfusion with pallor and mottled skin, metabolic acidosis, tachycardia or bradycardia, apnea, and respiratory distress) following full treatment, along with microbiological cure, which referred to a negative blood culture [13].

Blood cultures were performed using an automated blood culture system (BD BACTEC FX200, BD, Franklin Lakes). Bacterial identification and antimicrobial susceptibility testing were conducted using a fully automatic time‐of‐flight mass spectrometer system (VITEK MS, bioMérieux, Massy‐les‐Tours, France) and an automated identification and susceptibility testing system (BD Phoenix M50, BD, Franklin Lakes), respectively. Quality control was ensured using the reference strains *E. coli* ATCC 25922 and *Pseudomonas aeruginosa* ATCC 27853. The immunochromatographic assay (NG‐TEST CARBA5, Fosun Diagnostics, Shanghai, China) was used for carbapenemase testing, which detected *K. pneumoniae* carbapenemase (KPC), New Delhi metallo‐β‐lactamase (NDM), Verona integron–encoded metallo‐β‐lactamase (VIM), imipenemase metallo‐β‐lactamase (IMP), and oxacillinase‐48‐type carbapenemases (OXA‐48).

### 2.3. Statistical Analysis

Continuous variables with normal distributions are reported as means ± standard deviations. Nonnormally distributed data are presented as medians with interquartile ranges. Categorical variables are expressed as frequencies and proportions.

## 3. Results

### 3.1. Clinical Characteristics

A total of 9871 neonates were admitted to our hospital during the study period. Among them, 22 neonates were diagnosed with CRE BSIs, yielding an incidence rate of 2.2 cases per 1000 admissions (95% confidence interval: 1.4–3.4).

All 22 neonates had hospital‐acquired late‐onset infections (Table 1). Among them, males accounted for 77.3%, 81.8% were born prematurely (36.4% were extremely preterm infants), and 72.7% had a history of antimicrobial exposure, most commonly carbapenems (45.5%). Additionally, 72.7% underwent invasive procedures, of whom 54.5% underwent surgeries, including 10 intestinal surgeries, one esophageal atresia repair and gastrostomy surgery, and one choledochal cyst surgery.

**Table 1:** Clinical characteristics of the 22 neonates.

Variable	Values
Male sex, *n* (%)	17 (77.3)
Gestational age (weeks), mean ± SD	33.3 ± 3.73
< 28, *n* (%)	1 (4.5)
28–31^+6^, *n* (%)	7 (31.8)
32–36^+6^, *n* (%)	10 (45.5)
≥ 37, *n* (%)	4 (18.2)
Birth weight (g), median, and IQR	1525 (1247, 2520)
< 1000, *n* (%)	2 (9.1)
1000–1499, *n* (%)	9 (40.9)
1500–2499, *n* (%)	6 (27.3)
≥ 2500, *n* (%)	5 (22.7)
Age at onset of infection (days), median, and IQR	34 (15.5, 55.0)
Multiple births, *n* (%)	4 (18.2)
In vitro fertilization and embryo transfer, *n* (%)	2 (9.1)
Cesarean section, *n* (%)	19 (86.4)
Gestational diabetes mellitus, *n* (%)	4 (18.2)
Hypertensive in pregnancy, *n* (%)	6 (27.3)
Antimicrobial exposure, *n* (%)	16 (72.7)
Carbapenems, *n* (%)	10 (45.5)
β lactam/*β* lactamase inhibitors, *n* (%)	6 (27.3)
Invasive procedures before infection, *n* (%)	16 (72.7)
Central venous catheterization, *n* (%)	11 (50.0)
Mechanical ventilation, *n* (%)	3 (13.6)
Parenteral nutrition, *n* (%)	14 (63.6)
Surgery, *n* (%)	12 (54.5)

### 3.2. Microbiological and Carbapenemase Testing

Among the 22 isolates, *K. pneumoniae* was the predominant pathogen (20/22, 90.9%). The remaining two isolates were *E. coli* and *Klebsiella oxytoca*. Carbapenemase testing was conducted on a total of 18 isolates, among which 16 strains were isolated after 2020. Among the six bacterial strains isolated before 2020, two preserved strains were retrospectively tested. KPC (12/18, 66.7%) was the predominant carbapenemase, followed by NDM (6, 33.3%). Thirteen strains were simultaneously isolated from other sites, including synovial fluid, catheter tips, sputum, bone marrow, urine, surgical incisions, and ascitic fluid.

### 3.3. Antimicrobial Susceptibility

**Figure 1:**
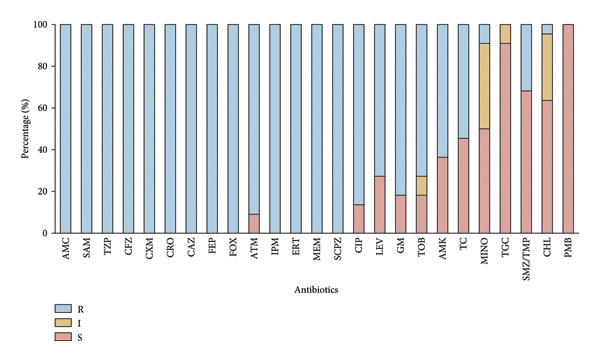
Antimicrobial susceptibility for the 22 isolates. R: resistant; I: intermediate; S: sensitive. AMC: amoxicillin/clavulanic acid; SAM: sulbactam/ampicillin; TZP: piperacillin/tazobactam; CFZ: cefuroxime; CXM: cefuroxime; CRO: cefixime; CAZ: ceftazidime; FEP: cefepime; FOX: cefoxitin; ATM: aztreonam; IPM: imipenem; ERT: ertapenem; MEM: meropenem; SCPZ: cefoperazone sulbactam; CIP: ciprofloxacin; LEV: levofloxacin; GM: gentamicin; TOB: tobramycin; AMK: amikacin; TC: tetracycline; MINO: minocycline; TGC: tigecycline; SMZ/TMP: sulfamethoxazole/trimethoprim; CHL: chloramphenicol; PMB: polymyxin B.

All *β*‐lactams exhibited 100% resistance rates except aztreonam (90.9% resistance; Figure 1). The resistance rate to levofloxacin was 63.6%. Most isolates (81.8%) were resistant to gentamicin, and 63.6% were resistant to amikacin. No isolates exhibited resistance to tigecycline or polymyxin (MIC ≤ 1).

### 3.4. Antimicrobial Therapy

All 22 neonates received empirical antimicrobial therapy (Table 2). Twenty neonates subsequently received definitive antimicrobial therapy following pathogen identification and achieved clinical cure after receiving full antimicrobial therapy, with a median duration of 17 days (interquartile range 11–26). Based on the carbapenemase detection results, antimicrobial agents were changed for 8 infants (Details in Table S1). Specifically, four neonates with KPC‐producing CRE infections received CAZ‐AVI (50 mg/kg q8h) alone, while two others with KPC‐producing CRE infections received CAZ‐AVI (50 mg/kg q8h) plus meropenem (40 mg/kg q8h). Two neonates with NDM‐producing CRE infections were treated with CAZ‐AVI (50 mg/kg q8h) combined with aztreonam (30 mg/kg q6h).

**Table 2:** Antimicrobial therapy data of the 22 neonates.

Antimicrobial therapy	*n* (%)
Empirical antimicrobial therapy (*N* = 22)	
Third‐generation cephalosporins	4 (18.2)
TZP	1 (4.5)
MEM	17 (77.3)
Definitive antimicrobial therapy (*N* = 20)	
MEM alone	8 (40.0)
PMB + MEM	3 (15.0)
CAV/AVI alone	4 (20.0)
CAV/AVI + MEM	2 (10.0)
CAV/AVI + ATM	2 (10.0)
ATM + MEM	1 (5.0)

*Note:* TZP: piperacillin/tazobactam; MEM: meropenem; PMB: polymyxin B; ATM: aztreonam; CAZ‐AVI: ceftazidime‐avibactam.

No severe adverse events occurred due to the use of antibiotics. Only one infant developed skin pigmentation, which was clearly linked to the use of polymyxin B. In long‐term follow‐up, the skin pigmentation gradually disappeared without leaving any long‐term effects. Three other neonates developed mild and temporary liver function abnormalities, and one experienced mild and temporary diarrhea, while the relevance between antibiotics and adverse events was classified as “likely” or “very likely.”

### 3.5. Concurrent Infections, Complications, and Outcomes

Half of the neonates developed concurrent infections at other sites (Table 3). Three neonates had meningitis as a complication, with cerebrospinal fluid white blood cell counts ranging from 41 to 63 × 10^6^/L. All cerebrospinal fluid cultures were negative. The median duration of hospitalization was 56 days (interquartile range: 27–71).

**Table 3:** Concurrent infections, complications, and outcomes of the 22 neonates.

Variable	*n* (%)
Concurrent infections	11 (50.0)
Peritonitis	5 (22.7)
Arthritis	4 (18.2)
Osteomyelitis	4 (18.2)
Meningitis	3 (13.6)
Urinary tract infection	2 (9.1)
Pneumonia	2 (9.1)
Complications	11 (50.0)
Septic shock	7 (31.8)
Necrotizing enterocolitis	4 (18.2)
Intraventricular hemorrhage	5 (22.7)
Intracerebral hemorrhage	2 (9.1)
Hydrocephalus	1 (4.5)
Death	3 (13.6)

Three neonates (13.6%) died, including Cases 1, 21, and 22 (Table S1). Case 1: A 30‐week preterm infant developed sepsis complicated by septic shock and necrotizing enterocolitis (Bell’s stage IIIB) on the 35th day of life. Unstable respiratory and heart rates, along with a low platelet count, made it difficult for the baby to withstand surgical intervention. Despite two days of empirical meropenem therapy (40 mg/kg q8h) and intensive support care, the infant died in the hospital before pathogen identification. Case 21: A 28‐week preterm infant developed CRE sepsis on the 33rd day of life. Although the neonate achieved clinical cure after effective antimicrobial therapy, treatment was discontinued due to severe intracranial hemorrhage, encephalomalacia, and hydrocephalus. The infant died immediately after discharge. Case 22: A 27‐week preterm infant developed necrotizing enterocolitis (Bell’s stage IIIB) complicated by intestinal perforation, sepsis, septic shock, and coagulopathy on the 11th day of life. Despite undergoing surgery and treating with meropenem and other supportive treatments for 2 days, the infant’s condition continued to worsen. Life support was withdrawn on the 13th day of life, and the infant died before pathogen identification.

## 4. Discussion

Enterobacteriaceae remain the predominant pathogens causing nosocomial infections in neonates, especially in developing countries. Here, we report 22 cases of neonatal CRE BSIs at a tertiary NICU in China and summarize the clinical experience of antimicrobial therapy in neonatal CRE BSIs.

### 4.1. Clinical Characteristics and Risk Factors

In this study, males accounted for a higher proportion of neonates with CRE BSIs than females (77.3% vs. 22.7%). Additionally, preterm infants faced an elevated risk of CRE BSIs, primarily due to their prolonged hospital stays and increased exposure to invasive medical procedures [14]. A study from Taiwan reported that preterm infants accounted for 66.7% (32/48) of children under 1 year of age with CRE infections [15]. Among the neonates enrolled in this study, 77.3% were preterm infants, consistent with previous findings [15].

Studies have reported that exposure to broad‐spectrum antibiotics is associated with multiple adverse outcomes, including MDR bacterial infections, necrotizing enterocolitis, and death [16]. Similarly, our study observed a high exposure rate (67.6%) to broad‐spectrum antibiotics among the enrolled neonates before the onset of CRE BSIs. Furthermore, 54.5% occurred following surgical procedures, which may have disrupted the neonatal immune barrier and substantially increased the risk of CRE infection [14].

### 4.2. Antimicrobial Therapy

The primary mechanism of carbapenem resistance in CRE is the production of carbapenemases, including Class A (such as KPC), Class B (such as NDM, IMP, and VIM), and Class D (such as OXA‐48) [17]. Different carbapenemases exhibit varying hydrolytic activities against different carbapenem antibiotics. Therefore, carbapenemase testing plays a crucial role in guiding CRE infection treatment and in developing prevention and control strategies [18]. The prevalence of carbapenemases varies over time and across different regions. A children’s hospital in Shanghai reported a shift in dominance from NDM to KPC between 2017 and 2021 [19]. Our study identified KPC as the predominant carbapenemase in our unit, accounting for 68.8% of enrolled neonates, while NDM accounted for 31.2%. OXA‐48 was not detected. This is the first report in China on the therapeutic decision making of neonatal CRE BSIs referring to carbapenemase testing.

CAZ‐AVI exhibits antibacterial activity against various *β*‐lactamases, including KPC, making it the first‐line treatment for adult CRE infections [5, 6]. According to CHINET data, the in vitro sensitivity of CAZ‐AVI against *K*. *pneumoniae* was as high as 84.6% [20]. In 2019, CAZ‐AVI was approved for clinical use in China. However, clinical experience with CAZ‐AVI in neonates remains limited. The successful use of CAZ‐AVI in eight neonatal CRE BSIs (including two cases of neonatal sepsis complicated by osteomyelitis caused by CRKP reported in 2021 [21]) in this study demonstrated its therapeutic effectiveness and safety. This aligned with previous reports [22] which demonstrate great potential of this agent in treating neonatal CRE infections.

Aztreonam is stable against hydrolysis by metallo‐β‐lactamases (MBLs) but remains susceptible to other non‐MBL *β*‐lactamases. The inhibition of non‐MBL enzymes by avibactam counteracts this vulnerability, resulting in synergistic activity with aztreonam against MBL‐producing CRE [23]. Capitalizing on this mechanism, the aztreonam‐avibactam was approved for treating severe CRE infections in adults in the European Union (2024) and the United States (2025). However, this drug was difficult to access in China. Therefore, we chose to use the combination of aztreonam and CAZ‐AVI as an alternative. In this study, two neonates infected with NDM‐producing CRE were treated with aztreonam combined with CAZ‐AVI, achieving favorable therapeutic outcomes without significant adverse effects. This is the first report of using this combination to cure neonates with MBL‐producing CRE infections. In 2024, a preterm infant in China with OXA‐48‐producing CRKP BSI was cured with aztreonam combined with CAZ‐AVI [24]. In 2023, an Indian preterm infant with CRKP (producing both NDM and OXA‐48) BSI was initially treated with polymyxin B, followed by CAZ‐AVI combined with aztreonam, but this regimen was ineffective. The infant was ultimately cured successfully with cefiderocol [25]. Besides this, the potential of certain novel antibiotic adjuvants to enhance the activity of antibacterial agents for treating MDR bacterial infections in neonates warrants further exploration [26].

Although current guidelines discourage meropenem monotherapy for treating CRE infections [5–7], clinical improvement was observed in eight neonates following empirical meropenem monotherapy in our study, thereby avoiding combination therapy. While existing evidence is limited to in vitro experiments and sporadic case reports [27, 28], high‐dose extended‐infusion meropenem remains effective in select neonates. A systematic review revealed that traditional meropenem monotherapy for treating CRKP with meropenem resistance (MIC > 8 mg/L) had a success rate of only 29.0% [29]. However, it also indicated that the success rate of meropenem monotherapy for meropenem‐intermediate isolates (MIC > 2 and ≤ 8 mg/L) could exceed 60%, approaching that of meropenem‐sensitive isolates [29]. This evidence suggests that in vitro resistance is not fully equivalent to in vivo treatment failure. Clinical improvement may indicate the in vivo effectiveness of meropenem treatment, which is particularly meaningful in resource‐limited settings where novel agents are unavailable.

Despite previous studies reporting a relatively high mortality rate [9], the overall mortality rate in this study was only 13.6%. For neonates who showed a poor response to antimicrobial treatment, we promptly adjusted the antimicrobial agents based on the results of antimicrobial susceptibility testing and carbapenemase testing. No serious adverse events occurred, and all neonates who completed full treatment achieved clinical cure. These findings indicate that early identification of infection and precise antimicrobial treatment can effectively improve the prognosis of neonates with CRE BSIs. Furthermore, novel antimicrobial agents and their combinations demonstrate promising therapeutic potential in some cases.

### 4.3. Limitations

This study has several limitations. First, it was a single‐center study with a limited sample size, which may not fully represent the entire neonatal population in the region. Second, the absence of follow‐up data hindered the evaluation of long‐term prognosis. Finally, only cases with positive blood cultures were included, potentially introducing selection bias by excluding cases with negative blood cultures.

## 5. Conclusions

Neonates with immature immunity are more susceptible to CRE BSIs. Beyond clinical efficacy and antimicrobial susceptibility testing, carbapenemase testing also plays a crucial role in the treatment decision making of neonatal CRE infections. Early identification of infection and precise antimicrobial therapy can significantly improve the prognosis of neonatal infections. Additional research involving large sample sizes, multicenter designs, and long‐term follow‐up is urgently needed.

**Nomenclature**
CRE: Carbapenem‐resistant Enterobacteriaceae
BSIs: Bloodstream infections
CRKP: Carbapenem‐resistant *K. pneumoniae*
NICU: Neonatal intensive care unit
MDR: Multidrug‐resistant
CAZ‐AVI: Ceftazidime‐avibactam
MIC: Minimum inhibitory concentration
KPC: *K. pneumoniae* carbapenemase
NDM: New Delhi metallo‐β‐lactamase
VIM: Verona integron–encoded metallo‐β‐lactamase
IMP: Imipenemase metallo‐β‐lactamase
OXA‐48: Oxacillinase‐48‐type carbapenemases
MBL: Metallo‐β‐lactamase

## Ethics Statement

The study was conducted in accordance with the guidelines of the Declaration of Helsinki and approved by the Ethics Review Board of Hebei Children’s Hospital. All data were anonymized to ensure patient confidentiality. Given the retrospective nature of the study, the requirement for informed consent was waived.

## Conflicts of Interest

The authors declare no conflicts of interest.

## Author Contributions

**Zelei Liu**: writing–original draft (lead), formal analysis (lead), visualization (lead), data curation (lead), and investigation (lead). **Xingpu Xu**: writing–original draft (equal) and data curation (lead). **Sanni Li**: writing–review and editing (equal) and investigation (equal). **Mei Li**: investigation (equal) and methodology (equal). **Jiancheng Jiao**: data curation (equal), formal analysis (equal), and investigation (equal). **Yinghui Guo**: writing–review and editing (equal), conceptualization (equal), and methodology (lead). **Li Ma**: writing–review and editing (lead), conceptualization (lead), and funding acquisition (lead). Zelei Liu and Xingpu Xu contributed equally to this article.

## Funding

This work was supported by the S&T Program of Hebei (20377778D and 21377709D).

## Supporting Information

Table S1: Demographic characteristics, antimicrobial therapy, and outcomes of 22 neonates. Note: M: male; F: female; MEM: meropenem; PMB: Polymyxin B; ATM: aztreonam; CAZ‐AVI: ceftazidime‐avibactam. Adverse effects of antibiotics: toxicity (hepatotoxicity, nephrotoxicity, and neurotoxicity), allergy, gastrointestinal symptoms (vomiting and diarrhea), electrolyte disorders, skin pigmentation, and superinfection. Relevance: certain, very likely, likely, and unlikely.

## Supporting information


**Supporting Information** Additional supporting information can be found online in the Supporting Information section.

## Data Availability

Due to ethical restrictions related to patient privacy, the raw data are not publicly available. However, anonymized data may be provided to qualified researchers upon reasonable request and written consent from Institutional Review Board. Requests should be directed to Li Ma (Email Address: 18503292173@163.com).
