# Supplementary material for: Carbapenem‐Resistant Enterobacteriaceae Bloodstream Infections in Neonates: Clinical Characteristics and Antimicrobial Therapy
Source: Can J Infect Dis Med Microbiol. 2025 Dec 20;2025:5544605. doi: 10.1155/cjid/5544605 (PMC12747061; doi:10.1155/cjid/5544605)
Supplement: Supplementary file 1 — Supporting Information Additional supporting information can be found online in the Supporting Information section. [file CJID-2025-5544605-s001.docx]

Table S1: Demographic characteristics, antimicrobial therapy, and outcomes of 22 neonates

| No. | Gender | Age of onset (d) | GA (w) | BW (g) | Species | Carbapenemase | Culture | Empirical  antimicrobial therapy | Definitive antimicrobial therapy | Duration of  antimicrobial  therapy（d） | Adverse effects of antibiotics | Relevance | Outcome |
| --- | --- | --- | --- | --- | --- | --- | --- | --- | --- | --- | --- | --- | --- |
| 1 | M | 35 | 30 | 830 | *Klebsiella pneumoniae* | NA | Blood | MEM | - | 2 | No | - | Death |
| 2 | M | 35 | 32^+3^ | 2880 | *Klebsiella pneumoniae* | NA | Blood | MEM | MEM (20mg q8h) | 9 | No | - | Cured |
| 3 | M | 66 | 29^+4^ | 1240 | *Klebsiella pneumoniae* | KPC | Blood | MEM | MEM (20mg q8h) | 16 | No | - | Cured |
| 4 | M | 26 | 39^+1^ | 3000 | *Klebsiella pneumoniae* | NA | Blood | MEM | MEM (20mg q8h) | 30 | Abnormal liver function | Likely | Cured |
| 5 | M | 19 | 39^+6^ | 1600 | *Klebsiella pneumoniae* | KPC | Blood, Joint fluid | Cephalosporins | PMB (150 000 U q12h) + MEM (40mg q8h) | 36 | No | - | Cured |
| 6 | F | 45 | 39 | 3400 | *Klebsiella pneumoniae* | NA | Blood | MEM | MEM (40mg q8h) | 9 | Diarrhea | Very likely | Cured |
| 7 | F | 11 | 34^+4^ | 2400 | *Klebsiella pneumoniae* | KPC | Blood, Joint fluid, Marrow | Cephalosporins | CAZ-AVI (50mg q8h) | 61 | No | - | Cured |
| 8 | F | 22 | 38^+4^ | 3200 | *Klebsiella pneumoniae* | NDM | Blood, Catheter tip | MEM | PMB (150 000 U q12h) + MEM (40mg q8h) | 20 | Abnormal liver function | Unlikely | Cured |
| 9 | M | 36 | 32^+4^ | 1270 | *Klebsiella pneumoniae* | KPC | Blood, Joint fluid | MEM | CAZ-AVI (50mg q8h) | 25 | No | - | Cured |
| 10 | M | 14 | 32 | 1440 | *Klebsiella pneumoniae* | KPC | Blood, sputum | MEM | PMB (150 000 U q12h) + MEM (40mg q8h) | 32 | Skin pigmentation | Certain | Cured |
| 11 | M | 126 | 35^+4^ | 1600 | *Klebsiella pneumoniae* | KPC | Blood, Catheter tip | MEM | MEM (20mg q8h) | 10 | No | - | Cured |
| 12 | F | 55 | 32^+2^ | 1600 | *Escherichia coli* | NDM | Blood, Urine | MEM | ATM(30mg q6h) + MEM (40mg q8h) | 18 | Abnormal liver function | Likely | Cured |
| 13 | M | 11 | 36^+5^ | 3150 | *Klebsiella pneumoniae* | NDM | Blood | MEM | MEM (40mg q8h) | 24 | No | - | Cured |
| 14 | M | 16 | 30^+4^ | 1300 | *Klebsiella pneumoniae* | KPC | Blood | MEM | CAZ-AVI (50mg q8h) | 24 | No | - | Cured |
| 15 | M | 41 | 34^+4^ | 2200 | *Klebsiella pneumoniae* | KPC | Blood, Secretions from the surgical incision | MEM | CAZ-AVI (50mg q8h) + MEM (40mg q8h) | 18 | No | - | Cured |
| 16 | M | 68 | 30 | 1250 | *Klebsiella pneumoniae* | KPC | Blood | MEM | CAZ-AVI (50mg q8h) + MEM (40mg q8h) | 16 | No | - | Cured |
| 17 | M | 112 | 33^+6^ | 1260 | *Klebsiella pneumoniae* | KPC | Blood, Sputum, Catheter tip | MEM | MEM (40mg q8h) | 11 | No | - | Cured |
| 18 | M | 5 | 35+5 | 1900 | *Klebsiella pneumoniae* | NDM | Blood | MEM | MEM (30mg q8h) | 15 | No | - | Cured |
| 19 | F | 17 | 29+6 | 1230 | *Klebsiella pneumoniae* | NDM | Blood, abdominal fluid | MEM | CAZ-AVI (50mg q8h) + ATM (30mg q6h) | 34 | No | - | Cured |
| 20 | M | 55 | 30+4 | 1450 | Klebsiella pneumoniae | KPC | Blood | Cephalosporins | CAZ-AVI (50mg q8h) | 15 | No | - | Cured |
| 21 | M | 33 | 28 | 760 | Klebsiella *oxytoca* | NDM | Blood, Sputum | MEM | CAZ-AVI (50mg q8h) + ATM (30mg q6h) | 14 | No | - | Death |
| 22 | M | 12 | 27+3 | 1100 | Klebsiella pneumoniae | KPC | Blood | MEM | - | 2 | No | - | Death |

Note: M: Male; F: Female; MEM: Meropenem; PMB: Polymyxin B; ATM: Aztreonam; CAZ-AVI: Ceftazidime-Avibactam. Adverse effects of antibiotics: Toxicity (hepatotoxicity, nephrotoxicity, neurotoxicity, et al), allergy, gastrointestinal symptoms (vomiting, diarrhea), electrolyte disorders, skin pigmentation and superinfection. Relevance: Certain, very likely, likely, unlikely.^1^

Reference

1.Ministry of Health of the People's Republic of China. Measures for the Reporting and Monitoring of Adverse Drug Reactions (Decree No. 81 of the Ministry of Health of the People's Republic of China) .Published May 4, 2011. Accessed March 20, 2025. https://www.nmpa.gov.cn/yaopin/ypfgwj/ypfgbmgzh/20110504162501325.html
